# Effect of Uncertainty-Aware AI Models on Pharmacists’ Reaction Time and Decision-Making in a Web-Based Mock Medication Verification Task: Randomized Controlled Trial

**DOI:** 10.2196/64902

**Published:** 2025-04-18

**Authors:** Corey Lester, Brigid Rowell, Yifan Zheng, Zoe Co, Vincent Marshall, Jin Yong Kim, Qiyuan Chen, Raed Kontar, X Jessie Yang

**Affiliations:** 1 Department of Clinical Pharmacy, College of Pharmacy, University of Michigan, 428 Church Street, Ann Arbor, MI, 48109, United States, 1 734-647-8849; 2 Department of Learning Health Sciences, University of Michigan School of Medicine, Ann Arbor, MI, United States; 3 Department of Industrial and Operations Engineering, College of Engineering, University of Michigan, Ann Arbor, MI, United States

**Keywords:** artificial intelligence, AI, human-computer interaction, decision-making, human factors, randomized controlled trial, clinical decision support, prediction, pharmacist, verification, drug development, drug, diagnosis, clinical decision support systems

## Abstract

**Background:**

Artificial intelligence (AI)–based clinical decision support systems are increasingly used in health care. Uncertainty-aware AI presents the model’s confidence in its decision alongside its prediction, whereas black-box AI only provides a prediction. Little is known about how this type of AI affects health care providers’ work performance and reaction time.

**Objective:**

This study aimed to determine the effects of black-box and uncertainty-aware AI advice on pharmacist decision-making and reaction time.

**Methods:**

Recruitment emails were sent to pharmacists through professional listservs describing a web-based, crossover, randomized controlled trial. Participants were randomized to the black-box AI or uncertainty-aware AI condition in a 1:1 manner. Participants completed 100 mock verification tasks with AI help and 100 without AI help. The order of no help and AI help was randomized. Participants were exposed to correct and incorrect prescription fills, where the correct decision was to “accept” or “reject,” respectively. AI help provided correct (79%) or incorrect (21%) advice. Reaction times, participant decisions, AI advice, and AI help type were recorded for each verification. Likelihood ratio tests compared means across the three categories of AI type for each level of AI correctness.

**Results:**

A total of 30 participants provided complete datasets. An equal number of participants were in each AI condition. Participants’ decision-making performance and reaction times differed across the 3 conditions. Accurate AI recommendations resulted in the rejection of the incorrect drug 96.1% and 91.8% of the time for uncertainty-aware AI and black-box AI respectively, compared with 81.2% without AI help. Correctly dispensed medications were accepted at rates of 99.2% with black-box help, 94.1% with uncertainty-aware AI help, and 94.6% without AI help. Uncertainty-aware AI protected against bad AI advice to approve an incorrectly filled medication compared with black-box AI (83.3% vs 76.7%). When the AI recommended rejecting a correctly filled medication, pharmacists without AI help had a higher rate of correctly accepting the medication (94.6%) compared with uncertainty-aware AI help (86.2%) and black-box AI help (81.2%). Uncertainty-aware AI resulted in shorter reaction times than black-box AI and no AI help except in the scenario where “AI rejects the correct drug.” Black-box AI did not lead to reduced reaction times compared with pharmacists acting alone.

**Conclusions:**

Pharmacists’ performance and reaction times varied by AI type and AI accuracy. Overall, uncertainty-aware AI resulted in faster decision-making and acted as a safeguard against bad AI advice to approve a misfilled medication. Conversely, black-box AI had the longest reaction times, and user performance degraded in the presence of bad AI advice. However, uncertainty-aware AI could result in unnecessary double-checks, but it is preferred over false negative advice, where patients receive the wrong medication. These results highlight the importance of well-designed AI that addresses users’ needs, enhances performance, and avoids overreliance on AI.

## Introduction

Artificial intelligence (AI) is becoming increasingly prevalent in health care with a wide range of applications such as drug development [1], computer-aided diagnosis and detection [2,3], and clinical decision-making [4]. In particular, AI-based clinical decision support systems (CDSS) can improve medication safety and reduce medication errors. CDSS have been shown to significantly improve medication use and safety in areas such as drug-drug interactions, inappropriate prescribing in older adults and pregnant women, over- and underprescribing, patient counseling [5,6], and patient outcomes (eg, increased medication adherence, lower blood pressure, decreased adverse events) [6].

Recent advances in AI modeling such as deep neural networks have resulted in CDSS that are “black-box” systems; black-box outputs do not provide insight into the model’s decision-making process or confidence in its decision [7]. To address the calls for increased transparency in medical AI predictions [8-10], developers have started using uncertainty-aware AI models. Uncertainty-aware AI models present the model’s uncertainty, or confidence in its decision, alongside its prediction [11], thus providing a metric for the user to assess the AI’s reliability [12]. CDSS reliability is an essential component of human evaluation of AI’s trustworthiness which determines the user’s acceptability of a technology [7]. While uncertainty-aware AI models increase transparency, additional knowledge is required to interpret the findings which may initially confuse users, leading to increased cognitive effort and degraded decision-making [13].

In addition to transparency, automation bias and aversion must be considered when developing CDSS. Automation bias occurs when users forsake their own expertise in favor of the AI’s advice [12,14]. The overreliance caused by automation bias may result in users missing AI-generated errors. Automation aversion causes users to rapidly and persistently lose trust due to an AI-generated error even when the AI’s overall performance exceeds humans [12]. Consequently, human errors are missed. An ideal human-centered AI tool should generate clinically valid decisions while fostering trust and avoiding overreliance on AI.

An unexplored avenue for AI-based CDSS is the medication verification process. Medication verification is a vital yet time-consuming visual check to ensure the contents of a filled medication vial match the prescribed medication. Despite pharmacists’ careful medication verification process, dispensing errors occur in 1.5% of all prescriptions [15]. Vigilance, or the ability to maintain focus and alertness over long periods, is essential for repeated tasks such as medication verification [16]. Pharmacists must remain alert and cognitively engaged [17]; yet, human vigilance wanes over time. Waning vigilance may be due to fatigue, cognitive overload, or the mundanity of repetitive tasks [18,19]. This is especially concerning in community pharmacies due to the increasing prescription volumes. In 2022, the average community pharmacy dispensed approximately 1215 prescriptions per week [20]. The increase in prescription volumes necessitates additional effort and time from pharmacists who spend between 30%‐48% of their time verifying medications [21,22].

Our team developed an AI prototype to assist pharmacists with the medication verification task, with the goal of reducing dispensing errors, improving patient safety, and decreasing pharmacists’ workload. The development of our AI tool using user-centered design principles is described in a study by Zheng et al [23]. The purpose of this paper is to determine the effects of black-box and uncertainty-aware AI advice on pharmacist decision-making and reaction time.

## Methods

### Overview

We previously developed 2 AI conditions to assist pharmacists with making decisions when dispensing medications. The “black-box” condition does not provide the user with insight into the AI’s certainty or predicted probability of a correct decision. The “uncertainty-aware” condition provides the user with an estimate of certainty from the model predictions. We tested these conditions in an experimental study using mock verification tasks with pharmacists.

### Trial Design

A crossover, randomized controlled trial was conducted from January 2023 to May 2023 with licensed pharmacists in the United States. Eligible participants were randomized to either the black-box AI or uncertainty-aware AI condition in a 1:1 manner. All participants completed 100 mock verifications with AI help and 100 mock verifications without AI help. The order of no help and AI help was randomized.

### Participants

Recruitment emails describing the study were sent to pharmacists through the Minnesota Pharmacy Practice-Based Research Network listserv and the University of Michigan College of Pharmacy Preceptor Network listserv. Interested individuals contacted the study team directly and completed a screening phone call. Eligible participants were licensed pharmacists in the United States who were at least 18 years old and had access to a laptop or desktop computer with a webcam. Pharmacists were excluded if they required assistive technology to use the computer, wore eyeglasses with more than 1 power, had uncorrected cataracts, intraocular implants, glaucoma, or permanently dilated pupils, or had eye movement or alignment abnormalities (eg, lazy eye, strabismus, and nystagmus).

### AI Input

We previously obtained a dataset of 432,734 images from a mail-order pharmacy that fills and ships prescriptions to all 50 US states [24]. The data contain 1 year’s worth of images of oral medications (ie, tablets and capsules) inside a prescription vial filled by a robot. The images were taken as the final step of an automated system using a robot to count pills into a vial, label the vial, take photos of the vial’s contents, and cap the vial. The dataset images are aligned with a National Drug Code (NDC) label and different attributes including color, shape, size in millimeters of pills, manufacturer, tablet scoring, and imprint. The number of images for each NDC ranges from 3 to 12,105 with a median of 540. There are 12 different colors of medications labeled in these images: white (182,178/432,734; 42.1%), yellow (53,296/432,734; 12.3%), pink (39,241/432,734; 9.1%), orange (30,846/432,734; 7.1%), multi-color (25,442/432,734; 5.9%), green (22,689/432,734; 5.2%), red (22,433/432,734; 5.2%), blue (20,704/432,734; 4.8%), brown (16,260/432,734; 3.8%), purple (13,609/432,734; 3.1%), turquoise (3090/432,734; 0.7%), and gray (2946/432,734; 0.7%). Seven different shapes are identified in the data: round (214,475/432,734;49.6%), oval (144,568/432,734; 33.4%), capsule (69,892/432,734; 16.2%), hexagon-6-sided (1912/432,734; 0.4%), triangle (1226/432,734; 0.3%), trapezoid (464/432,734; 0.1%) and pentagon-five sided (197/432,734; 0.04%).

### Interventions

The AI model in our study refers to a Bayesian neural network that predicts the dispensed pills’ NDC along with the uncertainty of the predictions. It is realized by applying the random dropout technique [25] to the ResNet-34 [26] convolutional neural network. Rather than simply predicting the probabilities of belonging to a specific NDC, the dropout technique enables the neural network to sample a set of possible predictions which we use to measure the uncertainty of the prediction. In our research, the model generated 50 potential probabilities for every image.

Fill accuracy is a dichotomous variable (correct fill or incorrect fill) determined by comparing the fill image to the reference image (ground truth). Matching fill and reference image pairs are labeled correct fills and mismatched pairs are labeled incorrect fills. This serves as the ground truth for the image pair. AI accuracy (good prediction or bad prediction) is a variable that indicates the AI’s accuracy in predicting the fill image’s NDC. A “good prediction” means the AI correctly identified the NDC, whereas a “bad prediction” incorrectly predicted the NDC. Good predictions always recommend the correct user action (accept or reject), whereas bad predictions typically result in ill-advised recommendations.

Each AI condition consisted of 100 trials with 76 trials containing correctly filled medications and 24 trials containing misfilled medications. For the correctly filled medications, the AI accurately recommended accepting (AI Accept) in 60 trials and erroneously recommended rejecting (AI Reject) in 16 trials. For the 24 trials containing misfilled medications, the AI accurately recommended AI Reject for 22 trials and erroneously recommended AI Accept for 2 trials. Three of the AI Reject trials contained an incorrect fill coupled with a bad prediction resulting in the correct recommendation (AI Reject). Although the AI’s predicted NDC was incorrect (ie, misidentified the fill image NDC), the AI renders the correct advice to reject the misfilled medication.

While the AI model’s overall accuracy is 98.46%, it was lowered to 79% for the experiment. Participants’ interaction with AI errors is a critical component of the research. Lowering the model’s accuracy significantly reduced the number of AI help trials needed to display the requisite number of AI errors. In the no-help condition, 76% of the medications were correctly filled.

The NDCs, the reference images, and the filled images were gathered from the correctly and incorrectly predicted images of the Bayesian neural network model. To eliminate potential confounding variables, each reference NDC was shown no more than twice throughout the experiment. To avoid loss of variation in colors, capsules, and oddly shaped pills while excluding blurry fill images, the team members carefully selected from the model-predicted images. Each image was reviewed for accuracy by comparing a reference image (ground truth) to the fill image.

There were 40 unique NDCs in the incorrect prediction file of the Bayesian neural network model. The NDCs, the reference images, and the images for cases involving bad AI predictions (AI approves the incorrect drug, and AI rejects the correct drug) were gathered from the incorrect prediction file. The NDCs and the reference images for cases involving good AI prediction for the incorrectly filled medication (AI rejects the incorrect drug) were gathered from the incorrect prediction file. The filled images were gathered from the correct prediction file.

**Figure 1.**
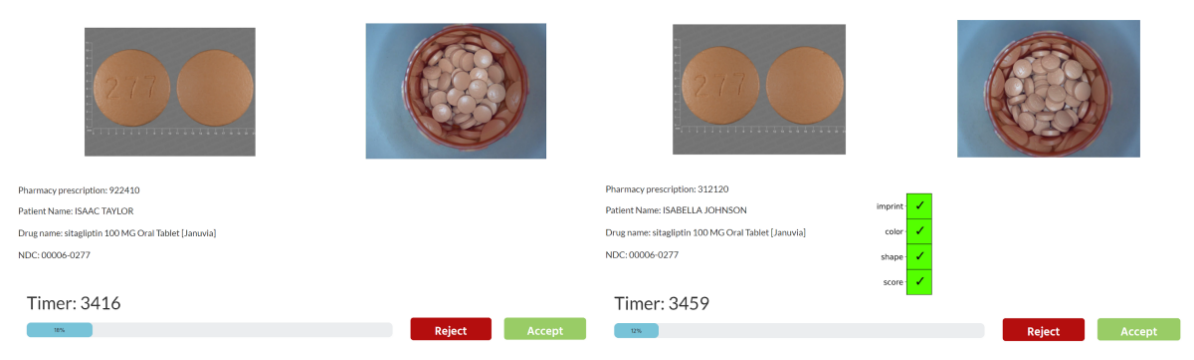
An example of the interface.

**Figure 2.**
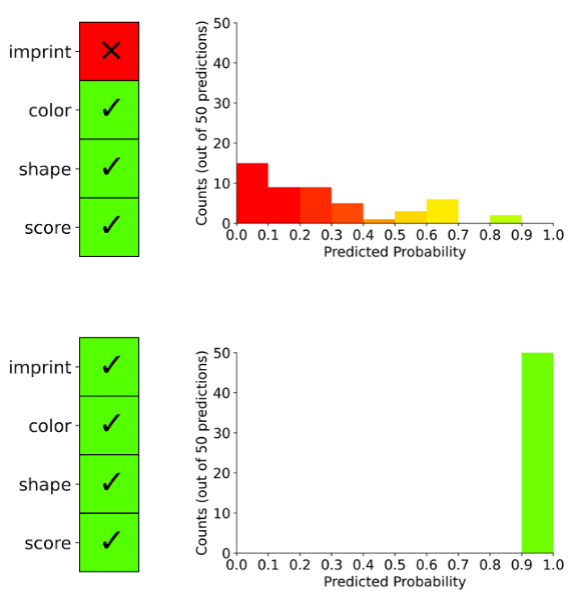
Presentations of 2 artificial intelligence (AI) models. The left-hand graphs are “black-box AI,” which displays “yes” or “no” for 4 pill characteristics: imprint, color, shape, and score. The right-hand graphs are “uncertainty-aware AI,” which illustrates the predicted probability of each set of predictions summarized in a histogram.

The study used Labvanced (Scicovery GmbH), an online, browser-based platform, to conduct the mock verifications. Participants met with a study team member through Zoom (Zoom Communications Inc) before completing the medication verification tasks to ensure technical and environmental requirements were met (eg, working webcam, adequate lighting, and quiet space). After confirming the participant’s setup was sufficient, the study team member provided the participant with a link to the experiment and a password. A video presentation explained how to perform the mock medication verification task using the study interface. Participants were informed that the goal of the task was to determine whether an image of a filled medication bottle matched a known reference image. The video presentation also explained the AI help condition to the participant with a tailored explanation based on randomization assignment. The interface displayed an image of a filled medication, a reference image, and prescription information (Figure 1). The task was to compare the “reference image” on the left to the “fill image” on the right to determine whether to accept or reject the dispensed medication. The “reference image” is the ground truth. For half the trials there was AI help on the bottom right of the screen. In the AI help conditions, the interface displayed AI advice as green checkboxes indicating AI matches based on characteristics of the pill (ie, black box), or a histogram with predicted probabilities (ie, uncertainty) and the checkbox figure (Figure 2). The AI advice was created from a ResNet-34 neural network.

Each participant completed 200 verification tasks, a block of 100 verifications with AI help, and a block of 100 verifications without AI help. The order of the AI help and no AI blocks was randomized for each participant to avoid an order effect, but the order of trials within each block was fixed. The pharmacist’s decision to approve or reject the filled medication and the time to decide were recorded for each verification. After each verification in the AI help condition, participants rated how much they trusted the AI advice on a scale of 0 to 100.

### Analyses

The outcomes of accuracy and reaction time were analyzed in mixed model generalized linear models with categorical predictors. The generalized linear models formulas were Performance~main.eff+(1|Participant_Nr) for accuracy and Reaction_Time~main.eff+(1|Participant_Nr) for reaction time. Categorical predictors included the AI type (ie, black-box AI, uncertainty-aware AI, or no AI help), and AI correctness versus ground truth, making the 2 × 2 comparison with 4 categories. Likelihood ratio tests compared means across the 3 categories of AI type for each level of AI correctness. The simple effects of uncertainty-aware AI and black-box AI and user performance based on AI type were examined. Statistical comparisons were performed with R statistical software (version 4.2.2, R Foundation for Statistical Computing) [27].

### Randomization

A random number generator in R software was used to ensure unbiased study designs. Numbers from 1 to 8 were randomly generated for each participant and participants had an equal probability of being assigned to either the uncertainty-aware AI condition or the black-box AI condition at the start. An equal number of participants were sought for each of the 8 study conditions.

### Ethical Considerations

The University of Michigan Institutional Review Board determined this research (HUM00213493) met the criteria for Exemption #3 and was exempt from institutional review board oversight. All participants signed a prospective agreement before any research activities. Participants received a US $150 check for completing the study. All study data have been deidentified. The experimental data were collected in Labvanced using a computer-generated unique 6-digit code.

## Results

### Recruitment

**Figure 3.**
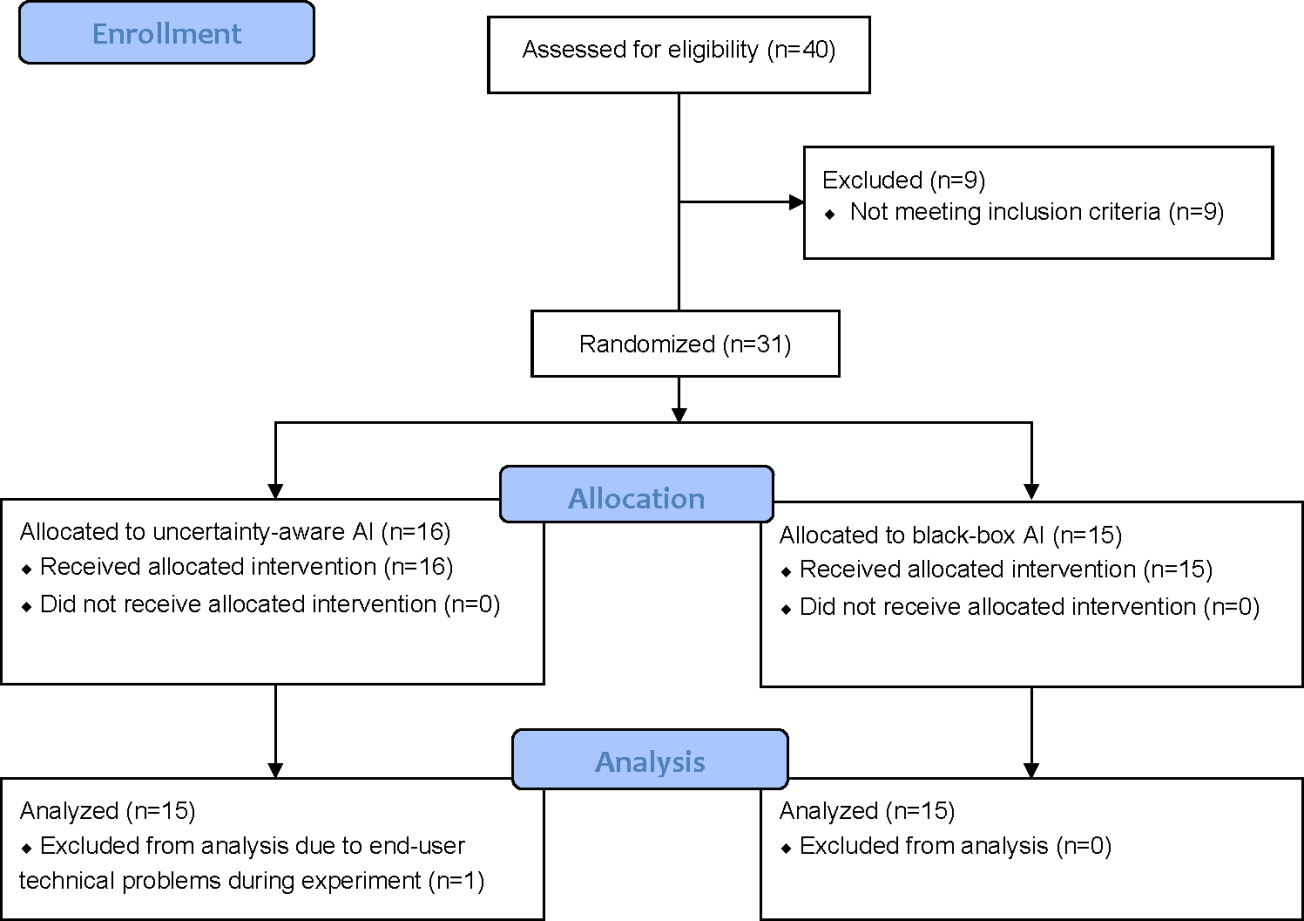
Participant flow of the randomized controlled trial for effect of AI models on medication dispensing in pharmacists. AI: artificial intelligence.

Recruitment began in January 2023 and the final participant visit was in May 2023. The single study visit was conducted remotely using a laptop or desktop computer. The trial ended when the target enrollment was met. In total, 40 pharmacists were assessed for eligibility. After excluding 9 pharmacists who did not meet the inclusion criteria, 31 participants were randomized for allocation in the “black-box AI” condition or the “uncertainty-aware AI” condition. Ultimately, 15 participants in each condition completed the experiment and were included in the subsequent analysis (Figure 3). The median length of the study visit was 59.3 (IRQ 53‐65.1) minutes.

### Study Population

As shown in Table 1, 30 pharmacists were included in the final analysis (black-box AI and uncertainty-aware AI condition). We investigated demographic characteristics for age, sex, race, practice settings, and working years. All the variables were well-balanced according to *P* values (*P*>.05). However, according to the effect sizes, some variables were unbalanced. Age had the largest value (0.68). Sex had the only true “small” effect size of 0.07 SD units. The rest were within the range of 0.07 and 0.42 SD units.

**Table 1.** Pharmacist demographics.

Characteristics	Overall (n=30)	Uncertainty-aware (n=15)	Black-box (n=15)	*P* value	Effect size	Interpretation
Age (year), median (IQR)	36 (32.25-45.75)	35.73 (9.68)	43.07 (11.79)	.07	0.68^a^	Medium
Sex (male), n (%)	13 (43.3)	7 (46.7)	6 (40)	>.99	0.07	Small
Ethnicity (non-Hispanic or Latino), n (%)	28 (93.3)	15 (100)	13 (86.7)	.46	0.27	Medium
**Race, n (%)**				.55	0.2	Small
Asian	5 (16.7)	3 (20)	2 (13.3)			
More than one race	1 (3.3)	0 (0)	1 (6.7)			
White	24 (80)	12 (80)	12 (80)			
**Practice setting, n (%)**				.25	0.42	Large
Community pharmacy	15 (50)	6 (40)	9 (60)			
Grocery store/mass merchandise pharmacy	1 (3.3)	1 (6.7)	0 (0)			
Hospital pharmacy	6 (20)	5 (33.3)	1 (6.7)			
Other	7 (23.3)	3 (20)	4 (26.7)			
Specialty pharmacy	1 (3.3)	0 (0)	1 (6.7)			
**Years worked (years), n (%)**				.34	0.33	Medium
1‐5	7 (23.3)	4 (26.7)	3 (20)			
6‐10	7 (23.3)	4 (26.7)	3 (20)			
11‐20	10 (33.3)	6 (40)	4 (26.7)			
21 or more	6 (20)	1 (6.7)	5 (33.3)			

a Effect size: Cohen’s *d*, the others are Cohen’s omega. Cohen’s omega chosen over phi coefficient due to zero cells.

### Outcomes

The effects of AI assistance on user trust are reported by Kim et al [28] In summary, pharmacists’ trust varied by AI help type and the verification outcome. Overall, uncertainty-aware AI significantly increased pharmacists’ trust.

**Figure 4.**
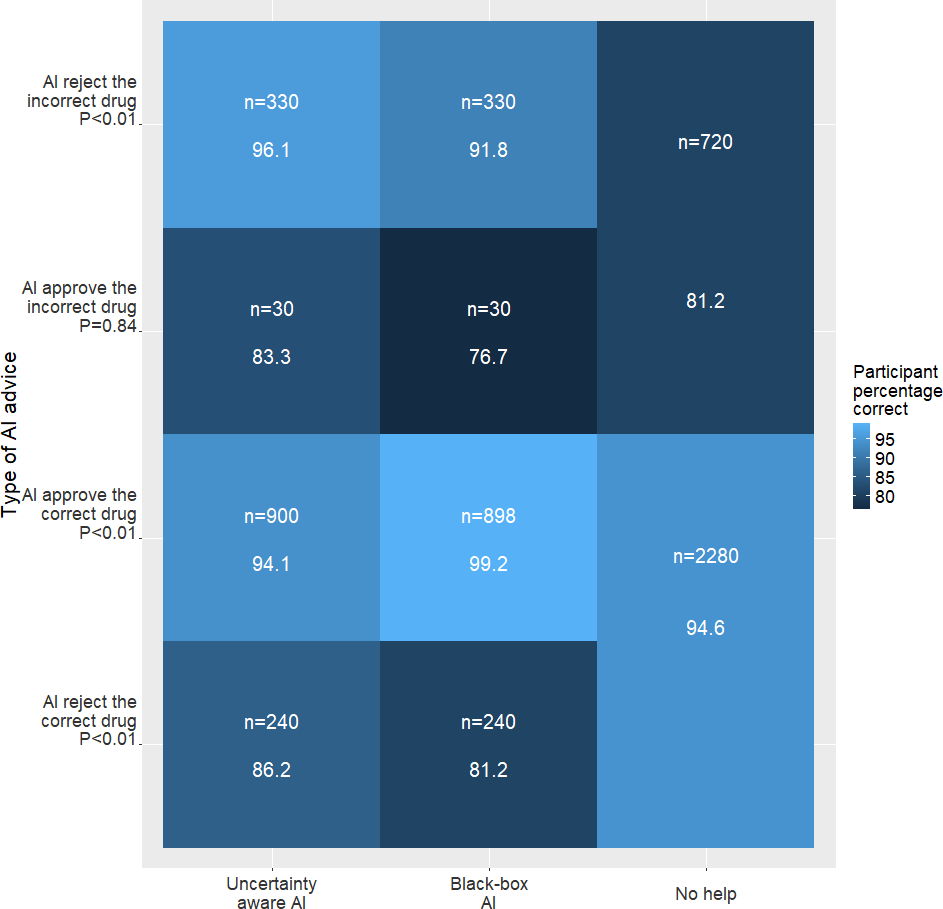
Plot of pharmacists’ decision-making performance (% of correct decisions; higher is better). AI: artificial intelligence.

**Figure 5.**
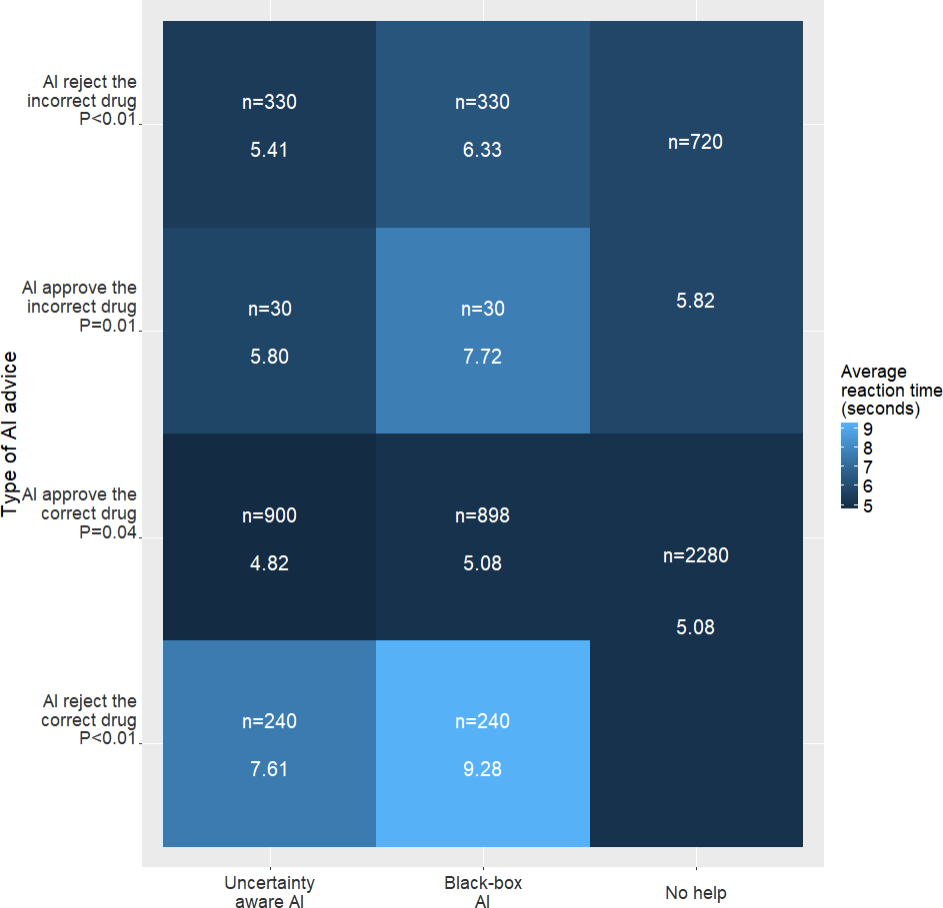
Plot of reaction time (in seconds; higher is worse) of included pharmacists. AI: artificial intelligence.

Pharmacists made the correct decision 91.3%, 93.1%, and 94.2% across all trials in the no-help, uncertainty-aware AI, and black-box AI blocks, respectively (*χ*^2^_2_=12.5, *P* value=.001). Performance differences emerged across the 3 conditions when examining performance by AI advice type. Figure 4 illustrates pharmacists’ performance based on AI accuracy (ie, good advice or bad advice). Similar differences were found for reaction time (Figure 5).

AI advice was divided into “Good advice” and “Bad advice.” For “Good advice,” 2 subtypes were identified: “AI Rejects Incorrect Drug” (Figure 4, Row 1) and “AI Approves Correct Drug” (Figure 4, row 3). In the first subtype, pharmacists rejected the incorrect drug 96.1% and 91.8% of the time for uncertainty-aware AI and black-box AI respectively, compared with no AI help (81.2%). When the AI suggested approving the correct drug, performances with black-box AI help surpassed those with uncertainty-aware AI help and no AI help (99.2% vs 94.1% vs 94.6%).

“AI Approves Incorrect Drug” (Figure 4, row 2) and “AI Rejects Correct Drug” (Figure 4, row 4) were classified in the Bad advice category. Despite AI’s incorrect predictions, pharmacists demonstrated superior outcomes with uncertainty-aware AI compared with black-box AI help. Uncertainty-aware AI helped protect against AI advice to approve an incorrectly filled medication in contrast with black-box AI (83.3% vs 76.7%). False alerts from AI to reject the correct drug, degraded pharmacists’ performance compared with baseline performance without AI help. Accuracy for no AI help, uncertainty-aware AI help, and black-box AI help was 94.6%, 86.2%, and 81.2%, respectively.

Figure 5 displays the reaction time for each condition. Pharmacists assisted by uncertainty-aware AI consistently exhibited shorter reaction times than those aided by black-box AI in the 4 scenarios discussed above (5.41 vs 6.33, 5.80 vs 7.72, 4.82 vs 5.08, 7.61 vs 9.28). Furthermore, the uncertainty-aware AI help condition showed improved reaction times compared with the no AI help condition except in the scenario where “AI rejects the correct drug.” Black-box AI did not lead to reduced reaction times in any of the situations as contrasted with no AI help.

## Discussion

### Principal Findings

In this study, we performed a randomized controlled trial to assess the effects of black box and uncertainty-aware AI tools on pharmacist decision-making and reaction time during the medication verification process. In our assessment of the impact of AI assistance on pharmacists’ reaction times, distinct patterns emerged between uncertainty-aware AI and black-box AI. Black-box AI increased reaction times compared with pharmacists acting alone. In contrast, pharmacists receiving uncertainty-aware AI advice had the quickest reaction times except when the AI erroneously recommended rejecting a correctly filled medication.

Research on the effectiveness of AI tools to improve efficiency in health care has shown mixed results. Wysocki et al’s [29] research showed that health care providers required significantly less time to make a decision when there was AI-user congruence with explainable AI assistance compared with the black-box AI assistance, which mirrors our results. However, in the face of health care provider-AI disagreement, no significant differences in time to decision were found between explainable AI and black-box AI [29]. A systematic review examining AI tools’ effects in safety-critical fields uncovered mixed results in AI’s ability to improve pathologists’ efficiency [30]. One study in the review showed pathologists with explainable AI assistance completed the task in only 35% of the time required for manual task completion [31], while another study found explainable AI assistance resulted in a significantly longer time to complete the task compared with pathologists working alone [32]. The variability of efficiency underscores the current state of affairs for AI in health care; the field is still in its infancy and more research is needed to understand how to balance transparency and interpretability. In our research with pharmacists, black-box AI’s inability to reduce reaction times in any of the scenarios prompts concerns about its practical efficacy in fast-paced pharmacy practice. Future research should examine the design of explainable AI visualizations to facilitate more immediate decision-making when the AI and user disagree.

Ensuring patients receive safe, accurate medication is the bedrock of the pharmacy profession. The pharmacist’s role mandates that the 5 rights of medication administration are upheld: right medication, right dose, right route, right patient, and right time [33]. Medication errors can result in significant patient harm, including emergency department visits, hospitalizations, and death [34-36] for which the pharmacist can be held legally responsible [37]. In safety-critical fields like medicine, the benefits of AI tools must be weighed against the risks [30]. In our study, the black-box AI facilitated greater acceptance of a correctly filled medication and thus, a reduction in unnecessary double-checks; however, this benefit is overshadowed by its propensity to misguide pharmacists to accept a potentially harmful misfilled medication. Crucially for this safety-critical field, the uncertainty-aware AI condition facilitated faster and more accurate identification of a misfilled medication than black-box AI or pharmacists acting alone. This demonstrates that uncertainty-aware AI safeguards against dispensing a misfilled medication. Gu et al’s [32] study of a human-AI diagnostic tool found pathologists preferred the AI system to err in the direction of false positives due to the harmful outcomes of missed diagnoses and delayed treatment. Similar to the medication verification process, pathologists’ reviews are performed quickly and pathologists indicated that the extra review is preferable to a missed diagnosis. Extrapolating these results to our study, uncertainty-aware AI’s ability to improve pharmacists’ identification of misfilled medications is preferable to the black-box AI help, and the potential workload increase due to unnecessary double-checks is offset by improved patient safety.

The histogram shown in the uncertainty-aware AI advice was developed based on pharmacists’ feedback during the user-centered design phase wherein they expressed a desire to see the probability of each predicted NDC to increase the model’s transparency [23]. In this case, the histogram may have attuned pharmacists to the idea of the AI advice being fallible and dispensing a wrong medication, whereas this factor was not apparent in the black box scenario. Pharmacists receiving uncertainty-aware advice were more likely to reject correctly and incorrectly filled medication regardless of the AI’s advice (accept or reject) suggesting the histogram introduced doubt which caused pharmacists to act more cautiously in order to avoid an incorrect dispense. Previous research has shown ambiguity in decision-making heightens an individual’s sensitivity to errors and results in more conservative behavior [38,39]. Pharmacists tend to be risk-averse individuals who dislike ambiguity [40], and when faced with ambiguity are likely to act conservatively (eg, reject a filled medication). In contrast, the black-box AI advice did not induce this same kind of doubt, perhaps leading pharmacists to “go with their gut.” Future research should explore more readily interpretable uncertainty visualizations to decrease ambiguity. A systematic review of 24 studies of human-AI collaboration in safety-critical fields found users prefer clean, simple, and easy-to-read visualizations [10]. Alternative ways to present AI uncertainty information to domain experts may balance transparency, uncertainty interpretability, and user performance.

Obtaining second opinions from colleagues is a common health care practice. AI can stand in for colleagues by quickly providing second opinions, but it is critical to communicate any ambiguity or uncertainty in its prediction to foster user trust and allow users to make judgments about when to trust the AI [8]. In interviews with pathologists about an AI tool, pathologists discussed the benefit of knowing their colleagues’ experiences, strengths, and weaknesses, thus allowing them to calibrate their responses to a colleague’s second opinion [38]. They identified needing a similar level of familiarity with the AI’s training, strengths, and weaknesses to successfully incorporate the AI’s advice in their decision-making process. Relatedly, previous research has shown that AI errors result in a significant and persistent loss of trust [41]. Explainable AI has been proposed as a solution to increase users’ trust by explicitly acknowledging the AI limitations [39]. However, Buçinca et al [42] found explainable AI may contribute to overreliance on AI advice. This contradicts our results from the uncertainty-aware condition; pharmacists with uncertainty-aware AI were more likely to reject a correctly filled medication when the uncertainty-aware AI suggested accepting it compared with black-box AI and the pharmacist acting alone. Conversely, our results indicate that black-box AI can be over-relied upon, especially false negative errors from the AI. A balance between fostering trust and avoiding overreliance is needed to ensure an optimal human-AI teaming experience.

The timing of AI advice relative to the user’s decision may influence the user’s performance and trust. Gajos and Mamykina [43] recently examined AI advice timing in 3 conditions: (1) AI recommendation and explanation before making a decision, (2) AI recommendation and explanation after making a decision, and (3) AI explanation only before making a decision. While all 3 conditions led to improved decision accuracy, only the third condition showed improvement in both decision accuracy and learning gain. A 2022 study of AI timing in clinical imaging found veterinary radiologists had lower trust in the AI, lower perceived utility of the AI, and less agreement with the AI, regardless of the AI’s correctness, when the advice was presented after their initial clinical decision [44]. Additional research should examine the timing of AI advice on pharmacists’ trust and accuracy.

### Limitations

The study has several limitations. First, the no help condition used a different image set than the AI help conditions. The same image set was used for both AI help conditions. While the images were randomly selected for both AI help and no help conditions, unanticipated biases in the image selection may exist. Second, the images were presented in a fixed order, which may have unintentionally biased the results. Future research should randomize the presentation of the images across all conditions for all participants to eliminate any inherent bias. Third, the small number of participants from 2 professional pharmacy networks may not be representative of the pharmacy community at large. Future research should be conducted with a broader, more diverse population of pharmacists. Finally, medication verification is only 1 step in the medication dispensing process. Additional research could examine the use of AI tools to support pharmacy staff at all steps of the dispensing process such as order entry, patient directions, and medication count.

### Conclusion

The effectiveness of AI assistance on pharmacists’ performance and reaction times varied by AI type and AI accuracy. Overall, uncertainty-aware AI resulted in faster decision-making, and black-box AI had the slowest decision-making. Concerningly, black-box AI worsened users’ accuracy when the AI provided bad advice, thus increasing the potential for patient harm. Uncertainty-aware AI acted as a safeguard against bad AI advice to approve a misfilled medication, reducing the chance of patient harm. However, pharmacists with uncertainty-aware AI performed worse and had longer reaction times when the AI recommended incorrectly rejecting a correctly filled medication compared with no AI help, leading to unnecessary double-checks. These results reinforce the importance of well-designed AI to meet users’ needs to ensure consistent benefits over no AI help.

## Supplementary material

10.2196/64902 Checklist 1 CONSORT-EHEALTH checklist (V 1.6.1).

## Abbreviations

AI: artificial intelligence
CDSS: clinical decision support systems
NDC: National Drug Code
